# Feasibility of Magnetic Resonance Fingerprinting on Aging MRI Hardware

**DOI:** 10.3390/tomography8010002

**Published:** 2021-12-23

**Authors:** Brendan Lee Eck, Kecheng Liu, Wei-ching Lo, Yun Jiang, Vikas Gulani, Nicole Seiberlich

**Affiliations:** 1 Imaging Institute, Cleveland Clinic, Cleveland, OH 44195, USA; 2 Department of Biomedical Engineering, Case Western Reserve University, Cleveland, OH 44106, USA; wxl317@case.edu (W.-c.L.); nse@med.umich.edu (N.S.); 3 Siemens Medical Solutions USA, Inc., Malvern, PA 19355, USA; kecheng.liu@siemens-healthineers.com; 4 Department of Radiology, University of Michigan, Ann Arbor, MI 48109, USA; yunjiang@umich.edu (Y.J.); vikasgulani@med.umich.edu (V.G.); 5 Department of Radiology, University Hospitals Cleveland Medical Center, Cleveland, OH 44106, USA

**Keywords:** magnetic resonance fingerprinting, accessible MRI, value MRI, quantitative MRI

## Abstract

The purpose of this work is to evaluate the feasibility of performing magnetic resonance fingerprinting (MRF) on older and lower-performance MRI hardware as a means to bring advanced imaging to the aging MRI install base. Phantom and in vivo experiments were performed on a 1.5T Siemens Aera (installed 2015) and 1.5T Siemens Symphony (installed 2002). A 2D spiral MRF sequence for simultaneous T_1_/T_2_/M_0_ mapping was implemented on both scanners with different gradient trajectories to accommodate system specifications. In phantom, for T_1_/T_2_ values in a physiologically relevant range (T_1_: 195–1539 ms; T_2_: 20–267 ms), scanners had strong correlation (R^2^ > 0.999) with average absolute percent difference of 8.1% and 10.1%, respectively. Comparison of the two trajectories on the newer scanner showed differences of 2.6% (T_1_) and 10.9% (T_2_), suggesting a partial explanation of the observed inter-scanner bias. Inter-scanner agreement was better when the same trajectory was used, with differences of 6.0% (T_1_) and 4.0% (T_2_). Intra-scanner coefficient of variation (CV) of T_1_ and T_2_ estimates in phantom were <2.0% and in vivo were ≤3.5%. In vivo inter-scanner white matter CV was 4.8% (T_1_) and 5.1% (T_2_). White matter measurements on the aging scanner after two months were consistent, with differences of 1.9% (T_1_) and 3.9% (T_2_). In conclusion, MRF is feasible on an aging MRI scanner and required only changes to the gradient trajectory.

## 1. Introduction

Recent advances in MRI data acquisition and reconstruction methods enable rapid scanning and multiparametric quantitative imaging [1,2]. Such techniques often require high-end MRI hardware, including high-performance gradient systems, exceptional field homogeneity, and receive coil arrays with a large number of channels. However, only a small portion of the world’s population has access to the newest scanner models capable of performing these advanced imaging techniques. Of the 32,000 MRI scanners installed globally, approximately 20% are over 10 years old [3], and the average age of an MRI scanner in the United States is 11.4 years [4]. High purchase price is a driver of continued use of aging MRI hardware. Reports have shown that the cost of a new 1.5T scanner is approximately $1.4M to $1.5M USD, with new ‘low-end’ scanners costing between $600,000 to $800,000 USD [5]. This large upfront investment in new hardware is prohibitive for many imaging centers worldwide, slowing the widespread use of advanced imaging. Additionally, the economic burden of the COVID-19 pandemic, estimated at over $16 trillion in the US [6], exerts even greater pressure on medical systems to reduce spending and maximize resource use. Deployment of advanced MRI techniques generally requires modern and high-performance MRI scanners in addition to significant investment in capital, skilled operators, and maintenance, consequently limiting access to tertiary care sites with funding and expertise. As such, there is a need for an MR imaging framework that can provide high-end, advanced imaging to existing scanners with their limited hardware set-ups across the install base.

Magnetic resonance fingerprinting (MRF) is a flexible framework that may enable advanced imaging on aging MRI scanners. While initially developed for simultaneous quantification of multiple tissue properties using the highest-end scanner hardware, MRF has features that may enable quantitative imaging on older or less-refined scanners. MRF does not require specific gradient trajectories beyond specified spatial resolution, field of view, and B_0_ field considerations [7,8], thus lessening the need for strong gradient systems; does not require parallel receive channels [9], thus enabling accelerated imaging even with few receive coils; can be used to correct for B_0_ [10] and B_1_ inhomogeneities [11], and thus may be useful even without ideal shim elements or B_1_ field shaping; and has the potential to eventually simplify protocols by enabling retrospective contrast synthesis from an MRF acquisition [12]. However, MRF has been predominantly developed at research centers with the latest MRI hardware, and thus its use on aging scanners has received limited investigation. In a recent report by Buonincontri et al., scanner hardware with 8–12 receive coil channels, 33–50 mT/m maximum gradient strengths, 120–200 T/m/s maximum slew rates, and four software versions were observed to provide reproducible T_1_ and T_2_ measurements in phantom and in the brain [13]. While these findings are encouraging, the feasibility of MRF has not been confirmed for scanners with less sophisticated hardware. On such older hardware, challenges including lower signal-to-noise ratio, poorer B_0_ homogeneity, and gradient imperfections (e.g., delays [14]) can result in MRF signals that may be difficult to interpret, requiring specialized data collection or tissue property map recovery approaches. The need for such modifications due to these potential sources of errors, and other unanticipated error sources, has not yet been investigated on aging MR hardware.

In this work, we explore the feasibility of performing MRF on aging MRI hardware (an 18-year-old, 1.5T scanner) as a step towards implementing this advanced technology on older and less powerful scanner platforms to democratize the deployment of this advanced technology. Our hypothesis is that, when modified to function on this system, MRF will provide comparable T_1_ and T_2_ maps in phantoms and in vivo across scanner platforms. To this end, the capabilities of the older scanner were first assessed, and the MRF sequence was modified according to scanner hardware constraints. Phantom and in vivo experiments were performed to assess inter-scanner agreement, repeatability, and reproducibility. While this work is a demonstration that MRF can be used on an older MRI system after modest adjustments of an implementation originally designed for a modern MRI system, our goal is to show that it may be possible to deploy MRF for high-value imaging on scanners designed to have more modest performance and a lower cost.

## 2. Materials and Methods

### 2.1. MRI Scanner Hardware

Data were collected on two different clinical MRI scanners. The more recently manufactured ‘modern’ scanner, located at the main campus of the institution and on which research MRF scans are routinely performed, is a 1.5T MAGNETOM Aera (Siemens Healthcare, Erlangen, Germany). This scanner, installed in 2015, will be referred to as the comparatively ‘new’ scanner. This scanner has a gradient system with maximum strength of 43 mT/m and maximum slew rate of 180 T/m/s. A 24-channel head coil available at the scanner was used. The ‘aging’ MRI scanner is located at a satellite site and used only for routine outpatient clinical scans prior to this study. This scanner, from here on referred to as the ‘old’ scanner, is a 1.5T MAGNETOM Symphony (Siemens). While this scanner was installed in 2002, it received a total imaging matrix (TIM) upgrade in 2015 (affecting only the receive coil system). The gradient system is weaker than on the ‘newer’ scanner, with a maximum strength of 30 mT/m and maximum slew rate of 100 T/m/s. A four-channel head coil available at the scanner was used in this study.

### 2.2. MRF Sequence

**Figure 1:**
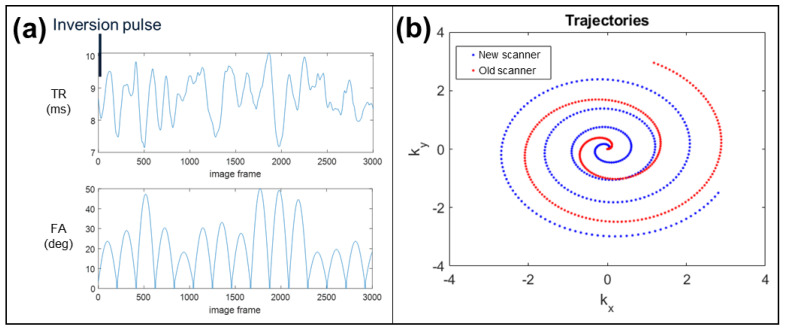
MRF sequence and k-space sampling trajectories. (**a**) For both scanners, the MRF sequence identically uses a variable repetition time (TR) and flip angle (FA) to induce sensitivity to T_1_ and T_2_ relaxation times. An inversion preparation pulse is applied prior to the first excitation. (**b**) Variable density spiral sampling trajectories are used on both scanners. The ‘weak gradient’ spiral designed for the old scanner MRF implementation (red) is more uniform than the ‘strong gradient’ spiral used for the new scanner MRF implementation (blue), resulting in a higher average acceleration factor (42 vs. 32, respectively).

The MRF sequence used for this study is based on fast imaging with steady state precession with spiral-out readout [15]. This sequence was implemented by the research team and is routinely deployed on the newer scanner used in this study. The sequence begins with an inversion pulse and uses variable repetition times (TR) and variable flip angles (FA) (Figure 1a). The radiofrequency excitation pulse used a Hanning-filtered sinc waveform with a duration of 2 ms and time-bandwidth product of 8 [15]. Echo times (TE) are held constant throughout the sequence, with TE = 2.5 ms. Data are collected for a single slice along a 48-arm variable density spiral trajectory [16], which fully samples the inner 50% of k-space in 24 spiral interleaves and the entire k-space in 48 spiral interleaves with an average overall acceleration factor of R = 32. A 3.75-degree incremental rotation pattern was used for spiral arms throughout the MRF acquisition train. The spatial resolution for this acquisition is 1.6 × 1.6 × 5.0 mm^3^ with a 400 × 400 mm^2^ field-of-view and duration of 26 s (3000 MRF frames) per imaging slice. Reconstruction is performed using the non-uniform fast Fourier transform [17], adaptive coil combination [18], and inner product based pattern matching to a dictionary to obtain T_1_ and T_2_ maps. The MRF dictionary included slice profile correction [19] and contained T_1_ values ranging from 10 to 4500 ms and T_2_ values ranging from 2 to 1000 ms (T_1_ values: 10 to 100 ms in 10 ms steps, 100 to 1000 ms in 20 ms steps, 1000 to 2000 ms in 40 ms steps, 2050 to 2950 ms in 100 ms steps, 3100 to 4500 ms in 200 ms steps. T_2_ values: 2 to 100 ms in 2 ms steps, 100 to 150 ms in 5 ms steps, 150 to 300 ms in 10 ms steps, 300 to 500 ms in 20 ms steps, 500 to 1000 in 50 ms steps). Proton density (M_0_) maps were computed based on the scaling of the measured signals relative to best-matched dictionary entries and normalized between 0 and 1.

In order to meet the hardware constraints of the ‘older’ scanner, modifications were made to the MRF acquisition. Due to the weaker gradients available on this system, either the trajectory from the new scanner could be used with a slower readout, or the trajectory could be modified such that the TR series could be kept the same. The latter approach was adopted so that the excitation scheme and MRF dictionary could be used without modification. This ‘weak gradient’ spiral trajectory designed for the old scanner, in contrast to the ‘strong gradient’ spiral described previously, uses a more uniform sampling density, requiring 48 interleaves to fully sample k-space and 40 interleaves to sample the inner 50% of k-space (for an average overall acceleration factor of R = 42). The spiral trajectories are shown in Figure 1b to demonstrate how they differ. Spiral trajectories were measured once on each scanner [20] and used to reconstruct corresponding MRF data. Upon implementation of the sequence on the old scanner, the TE was slightly different (TE = 2.2 ms). Other sequence parameters aside from those noted were unchanged. The same dictionary was used to reconstruct maps from the data collected on both scanners.

### 2.3. Phantom Experiments

The ISMRM/NIST MRI system phantom [21] was used to assess repeatability and reproducibility of the MRF-based measurements on both scanners. Documented reference T_1_ and T_2_ values are used to describe the phantom in following sections, whereas the measured T_1_ and T_2_ values on the new scanner were used for comparisons with the old scanner. The phantom was scanned five times on each of the scanners at a fixed position. Average T_1_ and T_2_ values in reference objects were measured and compared across scanners using Pearson’s correlation, Bland–Altman analyses, and inter-scanner coefficient of variation (CV). Additionally, the phantom was scanned on the new scanner using the ‘weak gradient’ spiral trajectory employed on the old scanner, and a Bland–Altman analysis was used to assess any differences in measurements made using the two different spiral trajectories with all other sequence parameters fixed. Bland–Altman analysis was used to also assess agreement between the new and old scanners using the same ‘weak gradient’ spiral. To assess intra-scanner measurement repeatability, CV was computed for each reference object. A test–retest experiment for the old scanner was performed on two different days to assess the reproducibility of T_1_ and T_2_ quantification.

### 2.4. In Vivo Experiments

Three healthy volunteers were recruited under an institutional review board approved protocol. Brain scans were obtained on both scanners. Five MRF scan repetitions were collected for each subject on each scanner at a fixed position. The ‘strong gradient’ spiral trajectory was used on the new scanner. Regions of interest (ROIs) were manually drawn by a biomedical engineer (B.E.) in the genu of the corpus callosum (white matter). ROI sizes were recorded. Average T_1_ and T_2_ values were recorded for each ROI. To assess intra-scanner repeatability, CV was computed using repeated scans on each scanner. The average and standard deviation of subjects’ intra-scanner CV was evaluated. To assess inter-scanner variability, CV was computed for each subject across scanners using the averaged T_1_ and T_2_ values from each scanner’s repeated measurements. The average and standard deviation of subjects’ inter-scanner CV is reported. To assess reproducibility on the old scanner, a test–retest scan was performed for one subject with two months between scans. White matter T_1_ and T_2_ values were compared for each subject between scanners and for one subject in a test–retest experiment using an unpaired, two-tailed Welch’s *t*-test with significance level of *p* = 0.05.

## 3. Results

### 3.1. Phantom Experiments

**Figure 2:**
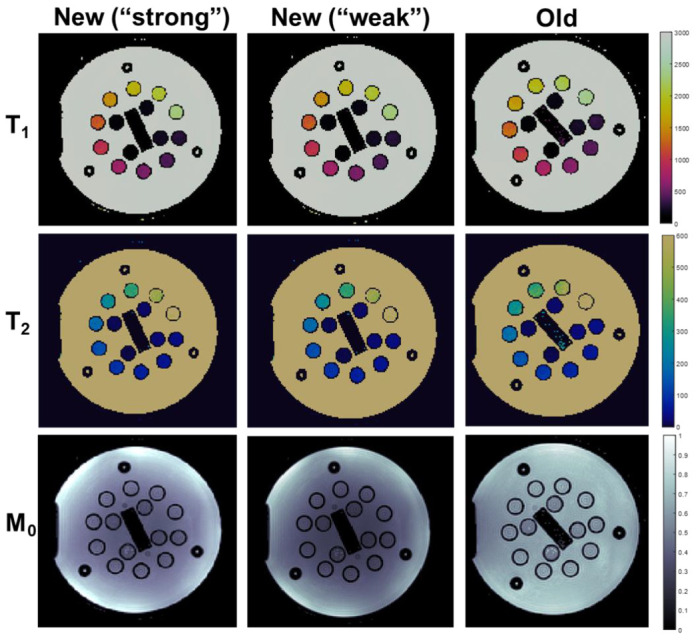
MRF maps of the ISMRM/NIST quantitative imaging phantom from the new scanner with the standard MRF spiral trajectory (‘strong’ gradients), the new scanner with the modified spiral trajectory (‘weak’ gradients), and the old scanner with the modified (‘weak’) spiral trajectory. T_1_ and T_2_ maps show comparable quality without large differences in reference object relaxometry values. M_0_ maps obtained from the two scanners have differing appearance potentially due to differences in receive coil sensitivities.

**Figure 3:**
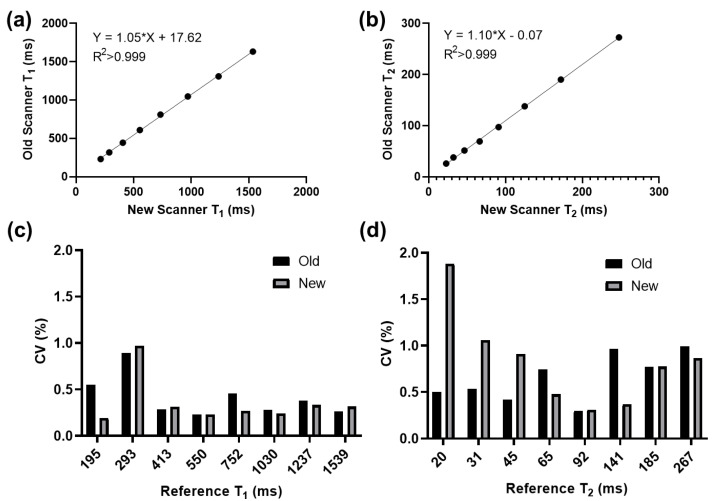
Correlation and repeatability analyses on the old scanner with the ‘weak gradient’ spiral and the new scanner with the ‘strong gradient’ spiral. (**a**,**b**) Correlation plots showing very strong inter−scanner correlation (*p* < 0.0001). Data points are averages from the five repeated acquisitions. (**c**,**d**) Bar plots of CV showing that the repeatability of T_1_ and T_2_ measurements (as computed from the five repeated scans) is comparable between the two scanners (<2% difference).

**Figure 4:**
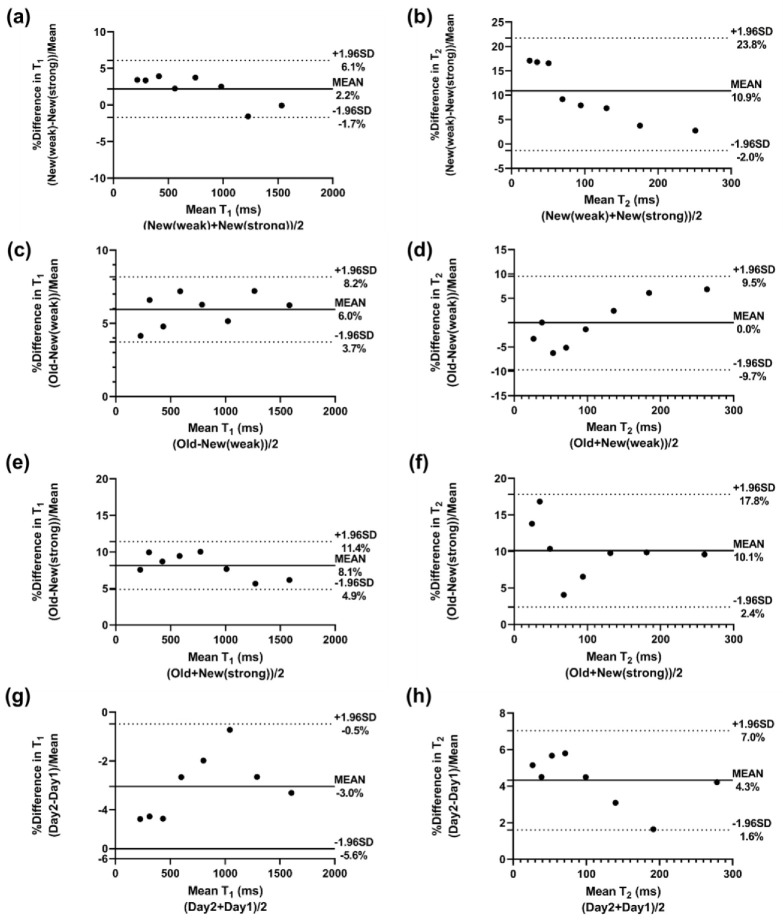
Bland–Altman analyses of T_1_ and T_2_ values obtained from the ISMRM/NIST MRI system phantom on the two scanners (old and new) showing patterns of bias and limits of agreement. (**a**,**b**) Comparison of the two evaluated spiral trajectories on the new scanner, the modified spiral with ‘weak’ gradients and the spiral conventionally used for MRF with ‘strong’ gradients. (**c**,**d**) Comparison of the new scanner with ‘weak gradient’ spiral versus the old scanner with the same ‘weak gradient’ spiral. (**e**,**f**) Comparison of the new scanner with ‘strong gradient’ spiral versus the old scanner with the ‘weak gradient’ spiral. (**g**,**h**) Comparison of T_1_ and T_2_ measurements on the old scanner across different days (test−retest assessment of reproducibility).

Representative MRF maps (T_1_, T_2_, M_0_) from both scanners are shown in Figure 2. T_1_ and T_2_ maps are qualitatively similar across scanners and spiral trajectories, whereas M_0_ maps exhibit different levels of spatial variation. The correlation and repeatability analyses are shown in Figure 3. Vials in the NIST phantom with T_1_ and T_2_ values within the physiologically relevant range of 195–1539 ms (T_1_) and 19.8–267.0 ms (T_2_) were analyzed. Measured values for vials outside this range were observed to be unstable on both scanners. Within the relevant range, the correlation between scanners for both T_1_ and T_2_ measurements was strong (R^2^ > 0.999, *p* < 0.0001). The intra-scanner CV was <1.0% for T_1_ and <2.0% for T_2_ in both scanners for vials with physiologically relevant values. Bland–Altman plots are shown in Figure 4. When comparing acquisitions on the new scanner with the ‘strong gradient’ and ‘weak gradient’ spiral trajectories, T_1_ differed by an average absolute percent difference of 2.6% and T_2_ by 10.9%. Comparison of the old and new scanners both with the ‘weak gradient’ spiral showed average absolute percent differences of 6.0% in T_1_ and 4.0% in T_2_. However, when comparing the old scanner to the new scanner with the ‘strong gradient’ spiral, T_1_ differed by an average of 8.1% and T_2_ by 10.1%. The average inter-scanner coefficients of variation between the old scanner and the new scanner with the ‘strong gradient’ spiral were 5.8% (T_1_) and 7.1% (T_2_). The results of the test–retest analysis on the old scanner showed strong correlation of T_1_ and T_2_ (R^2^ > 0.999, *p* < 0.0001) with an average T_1_ difference of 3.0% and T_2_ difference of 4.3%.

### 3.2. In Vivo Experiments

**Figure 5:**
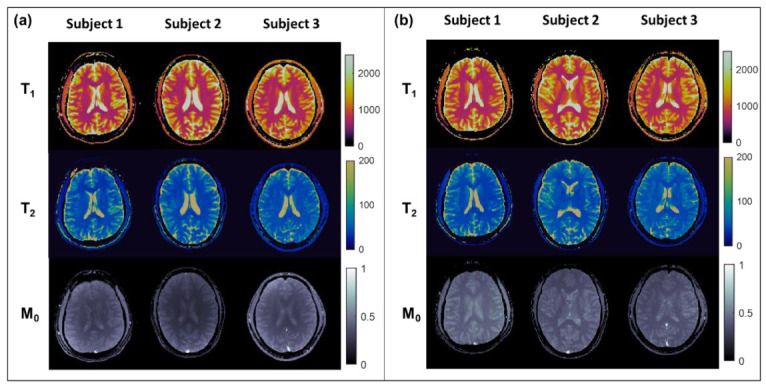
MRF maps of the brain from healthy subjects obtained from the new scanner (**a**) and the old scanner (**b**). T_1_ and T_2_ maps are of comparable overall quality, and values appear visually similar between corresponding tissue types. As also observed in phantom, M_0_ maps differ in appearance between the two scanners, potentially due to differences in receive coil sensitivities.

Representative MRF maps for the three subjects are shown in Figure 5. The average, standard deviation, intra-scanner CV, and inter-scanner CV for white matter ROIs are shown in Table 1. The mean and standard deviation of the ROIs sizes were 137 ± 17 mm^2^. The average differences of MRF values derived from the old scanner relative to the new scanner for the subjects were −23 ms (−3.1%) for T_1_ and +2.5 ms (+7.7%) for T_2_. Differences between the test–retest values on the old scanner were: +11 ms (+1.9%) for T_1_ and −1.5 ms (−3.9%) for T_2_. The intra-scanner coefficients of variation for the old and new scanners, respectively, were 2.9% and 2.1% for T_1_, and 2.6% to 3.5% for T_2_. The inter-scanner CV was 4.8% for T_1_ and 5.1% for T_2_.

## 4. Discussion

These findings demonstrate the feasibility of collecting tissue property maps using MRF on aging, lower performance MRI scanners. T_1_ and T_2_ maps obtained by MRF on both new and old scanners were of comparable appearance despite differences in gradient systems, number of receive channels, and software versions. Quantitative T_1_ and T_2_ measurements were repeatable, with intra-scanner CV of <2.0% for physiologically relevant phantom values (T_2_ between 20–267 ms) and CV of ≤6% for all subjects. Inter-scanner correlation of T_1_ and T_2_ was strong in phantom experiments (R^2^ > 0.999) with inter-scanner CV of 4.8% (T_1_) and 5.1% (T_2_). Reproducibility of in vivo T_1_ and T_2_ values in a single subject after two months was also good, with measurements within 4%.

MRF repeatability and reproducibility on the aging scanner were within the ranges anticipated based on previous reports. A study of MRF repeatability across 34 days for the ISMRM/NIST phantom showed CV <5% for all reference T_1_ and T_2_ values except T_2_ < 13 ms [21]. In the present study, phantom CV values for the physiologically relevant range of T_1_ and T_2_ were all similarly <5%. A multi-site study by Buonincontri et al. examined reproducibility in phantom and in vivo brain at 1.5T and 3T for scanners with 8–12 receive coil channels, 33–50 mT/m maximum gradient strengths, 120–200 T/m/s maximum slew rates, and four different software versions [13]. Authors reported intra-site white matter CV at 1.5T of 1.9–2.1% (T_1_) and 2.9–4.7% (T_2_), comparable to the CV of 2.1–2.9% (T_1_) and 2.6–3.5% (T_2_) observed in this work. However, the inter-scanner CV in the NIST phantom presented in this work, with averages of 5.8% (T_1_) and 7.1% (T_2_), was greater than that found by Buonincontri et al., 0.9% (T_1_) and 2.7% (T_2_).

MRF was successfully deployed on the old scanner to generate in vivo T_1_ and T_2_ maps similar in appearance to those collected on the new scanner. While there were statistically significant differences in white matter measurements between scanners for each subject, overall inter-scanner white matter CV in this work, 4.8% (T_1_) and 5.1% (T_2_), was within the inter-site variability reported by Buonincontri et al., 6.6% (T_1_) and 9.7% (T_2_). T_1_ values were lower than the overall white matter values reported by Buonincontri et al., but in agreement with previously reported T_1_ for genu of the corpus callosum at 1.5T (see Table 1 of [22]). T_2_ was underestimated as compared to previously reported values for the genu of the corpus callosum (see Figure 5 of [23]), which may be due to spoiler or magnetization transfer effects not accounted for in the dictionary generation [24,25]. Although inter-scanner variation was larger in the phantom studies, these findings suggest that T_1_ and T_2_ measurements on the aging scanner for white matter are within the inter-scanner variability reported in the literature. Note that the performance of the aging scanner in this work is comparable to that described in other work despite its reduced number of receive channels and weaker gradient system, both in maximum gradient strength and slew rate. Additionally, the scanners evaluated in the present study are of a different vendor than that evaluated by Buonincontri et al., suggesting that the repeatability and reproducibility of MRF are similar across scanner specifications and vendor.

Although MRF sequences were purposely designed to be as similar as possible when considering system limitations, factors in sequence design can influence parameter measurements. For example, it has been suggested that measured parameter values are affected by gradient trajectory. In cardiac MRF, a rosette trajectory with inherent fat suppression was shown to yield 2–3 ms higher T_2_ relaxation times compared to spiral MRF [8]. Differences in spiral trajectories in the present study may have contributed to the observed inter-scanner bias. The phantom experiment using both ‘weak gradient’ and ‘strong gradient’ spirals on the new scanner suggests that changing the spiral did indeed impact parameter estimates; T_2_ varied by approximately 10% when using the ‘weak’ spiral, comparable to the T_2_ differences observed when comparing the old scanner to the new scanner with the ‘strong gradient’ spiral. Furthermore, the comparison between scanners with the same ‘weak gradient’ spiral showed a smaller average absolute percent difference in the measured T_2_ values, below that observed in the old scanner test–retest comparison (4.0% vs. 4.3%). The difference between T_1_ measurements made on different scanners with the same ‘weak gradient’ spiral trajectory was smaller than inter-scanner T_1_ measurements made with different spirals (6% vs. 8%), although the remaining bias was larger than that observed in the test–retest experiment (6% vs. 3%). Differences in the undersampling patterns for the tested spiral trajectories may have contributed to these observed differences. It has been reported that T_1_ and T_2_ maps can have mean relative differences of up to 16.9% (T_1_) and 19.9% (T_2_) in the brain [26], and that these differences were significantly reduced by adjusting the spiral interleaving pattern and using an iterative reconstruction approach. Trajectories aside from spiral may be preferable on aging hardware, or the use of additional corrections or more complex patttern matching approaches (i.e., iterative reconstructions) may be required. It is possible that reduced SNR due to fewer receive channels in the aging scanner relative to the newer scanner may have also contributed to observed bias, which may need to be compensated by the use of these more advanced matching methods as previously reported [27]. FInally, there was a difference in TE for the sequence implementations on the two scanners that may have contributed to the small but observed differences in estimated T_1_ and T_2_ relaxation times.

Computation time and cost of computing is a consideration for deployment of MRF on aging MR hardware. Image reconstructions in this study were performed offline using a research workstation with an 18-core Intel i9-9980XE processor and 64 GB of RAM, although these specifications were well beyond the minimum resources necessary for reconstruction. Reconstruction times were within 1–2 min using non-optimized, in-house developed reconstruction code with a pre-computed MRF dictionary. Reports have shown that similar MRF reconstructions can be performed online using open-source Gadgetron tools [28]. Thus, the framework for implementing MRF with online reconstruction presently exists at a computer hardware cost of a few thousand US dollars, and potentially as low as hundreds of dollars. However, there are regulatory requirements for computing equipment that may limit the hardware available for clinical use, and the route of vendor approved upgrades may be costly. An alternative to on-site computing could be cloud computing, which has been previously demonstrated for compressed sensing reconstruction in under 5 min at a cost of $50/h and with a connection speed of 50 MB/s [29].

Although this work focused on implementation of MRF on aging hardware, MRF could potentially enable robust advanced imaging to be performed on MRI scanners designed to be less powerful and thus less expensive. As suggested from this work, reduced gradient specifications do not significantly alter the measurements made using MRF; designing a modern MRI scanner with weaker and slower gradients could consequently lead to cost savings both in scanner construction, as well as in electrical power and cooling infrastructure [5]. Similarly, because MRF does not require arrays of receiver coils, coil arrays and the receiver chain could be purposefully pared down to lower costs. Reduced field strength could also be a target for cost reduction, as 3D MRF of the brain has been recently demonstrated on a 0.55T system [30], and 3D MRF has even been demonstrated in a preliminary report at the ultra-low field of 50 mT using a Halbach system [31]. MRF may be used to loosen the relatively stringent field homogeneity requirements, as imperfections could be incorporated into the MRF acquisition and reconstruction framework. Inclusion of B_1_ mapping has been demonstrated to improve imaging in the presence of metallic objects [32] and gradient delays have been estimated and corrected as part of the MRF reconstruction [33]. The flexibility in hardware enabled by MRF could allow for lower-cost systems to be used in place of scans that are currently performed on a typical 1.5T or 3T scanner, enhancing the value and accessibility of MRI. Additionally, in such lower-cost systems or on existing hardware, MRF may serve as a ‘push button’ technique to simplify protocols and reduce dependence on highly skilled operators. Provided the hardware is sufficient, MRF can take advantage of system properties and features during data acquisition and image reconstruction, thus allowing acquisition to be adequately guided by even a junior technologist.

There are notable limitations of this study. The sample size for in vivo experiments is small, with only three subjects. An expanded study of more volunteers should be performed to identify systematic bias or differences in precision when performing MRF on the aging MRI scanner. MRF scans should be performed in patients with pathology on the aging scanner to determine clinical translation potential. Additionally, the aging scanner that was used did receive a “TIM” upgrade which, although it did not affect the gradients or main magnetic field, may have improved receive system performance as compared to a true original model scanner. M_0_ maps were not quantitatively analyzed in this work and were observed to have visually apparent differences between systems (e.g., Figure 2 and Figure 5). Given that M_0_ values are determined by the scaling of measured signal relative to the best-match dictionary signal, both the underlying proton density and coil sensitivities will contribute the measurement. The scanners in this work had significantly different receive coil configurations (4- vs. 24-channel), which likely contributed to observed differences. Last, system imperfections that might have contributed to the observed biases in T_1_ and T_2_, such as field inhomogeneity, as well as differences in sequence implementations across different hardware and software platforms such as TE in this work, should be further explored.

## 5. Conclusions

In conclusion, MRF is feasible on older scanners with weaker gradient systems and a small number of receiver coils. Overall repeatability and reproducibility of quantitative T_1_ and T_2_ was comparable to that previously observed in the literature; however, subtle differences in the MRF sequence or scanner characteristics may have contributed to bias in measured values. Findings suggest that newer data collection and processing approaches like MRF may enable older scanners to be used for state-of-the-art imaging, and may potentially drive the development of new, lower-cost MRI systems.

## Figures and Tables

**Table 1:** Mean and standard deviation of T_1_ and T_2_ values of white matter obtained from the old and new scanners for each subject as well as intra-scanner and inter-scanner coefficients of variation. *p*-values correspond to comparisons of T_1_ and T_2_ values within subjects across scanners (shown in columns) and in test–retest (shown in row 1 (p)). Significant differences (*p* < 0.05) are boldfaced.

Subject	T_1_ (Old)	T_1_ (New)	*p*	T_2_ (Old)	T_2_ (New)	*p*
**1**	599 ± 22	639 ± 10	**0.01**	37.0 ± 0.4	35.7 ± 0.5	**<0.01**
**1 (retest)**	610 ± 20			35.5 ± 1.1		
**1 (p)**	0.42			**0.03**		
**2**	626 ± 17	594 ± 14	**0.01**	36.1 ± 1.5	30.7 ± 1.7	**<0.01**
**3**	638 ± 15	697 ± 16	**<0.01**	37.6 ± 1.0	36.8 ± 1.3	0.38
**CV (intra)**	2.9%	2.1%		2.6%	3.5%	
**CV (inter)**	4.8%			5.1%		

## Data Availability

The data presented in this study are available upon reasonable request to the corresponding author. The data are not publicly available due to the sensitive nature of medical image data.
